# *Angelica dahurica* Exosome-like Nanovesicles Delivered by Ultrasonic Nebulization Alleviate Allergic Rhinitis and Modulate NF-κB Signaling

**DOI:** 10.3390/pharmaceutics18091193

**Published:** 2026-09-21

**Authors:** Anqi Zhou, Xiao Li, Lin Zhao, Song Zhang, Xiaowei Li, Qiulin Yue, Xin Sun, Kai Zheng, Le Su

**Affiliations:** 1 State Key Laboratory of Biobased Material and Green Papermaking, School of Bioengineering, Qilu University of Technology (Shandong Academy of Sciences), Jinan 250353, China; 19153557259@163.com (A.Z.); lixiao11262023@163.com (X.L.);; 2 Shandong Jicui Biotechnology Co., Ltd., Jinan 250100, China; 3 Institute of Environment and Ecology, Tsinghua Shenzhen International Graduate School, Shenzhen 518055, China; 4 Shengsheng Xiangrong Biotechnology (Shandong) Co., Ltd., Jinan 250002, China

**Keywords:** allergic rhinitis, *Angelica dahurica*, ultrasonic atomization, exosome-like nanovesicles, inflammatory factors

## Abstract

**Background:** Current treatments for allergic rhinitis (AR) are associated with side effects and do not provide a definitive cure. *Angelica dahurica* has recognized anti-inflammatory activity; however, the therapeutic potential of *Angelica dahurica*-derived exosome-like nanovesicles (ADELNs) in AR remains insufficiently explored. In addition, although ultrasonic nebulization is widely used for airway inflammatory diseases, including pulmonary disorders and asthma, its application in AR has received limited attention. **Methods:** ADELNs were isolated by differential centrifugation and ultracentrifugation and characterized using transmission electron microscopy (TEM), nanoparticle tracking analysis (NTA), dynamic light scattering (DLS), thin-layer chromatography (TLC), SDS-PAGE, and agarose gel electrophoresis. To evaluate the effects of ultrasonic nebulized ADELNs on AR, we designed and validated a mouse nasal nebulization device. Cellular inflammation and OVA-induced AR mouse models were then established, and inflammatory and pathological indicators were assessed using ELISA, RT-qPCR, histological staining, and related assays. Western blotting was used to investigate the involvement of the NF-κB signaling pathway. **Result:** ADELNs were successfully isolated and exhibited typical exosome-like characteristics. The ultrasonic nebulization device showed good stability. Nebulized ADELNs alleviated AR symptoms in mice in a concentration-dependent manner, reduced inflammatory cytokines, serum histamine, and OVA-specific IgE, downregulated inflammatory cytokine mRNA expression in the nasal mucosa, improved nasal mucosal thickness, and decreased mast cell infiltration. To explore the potential mechanism, ADELN treatment was associated with reduced IκB and p65 phosphorylation and decreased NF-κB nuclear translocation, suggesting that modulation of NF-κB signaling may contribute to the anti-inflammatory effects of ADELNs. **Conclusions:** This study successfully isolated and characterized ADELNs. Using a self-designed ultrasonic nebulization device for mouse nasal delivery, we showed that ADELNs inhibited cellular inflammation in vitro, alleviated symptoms and pathological injury in AR mice, and may exert anti-AR effects by suppressing the NF-κB signaling pathway. These findings provide a new strategy for AR treatment.

## 1. Introduction

Allergic rhinitis (AR) is a chronic, noninfectious inflammatory disease of the nasal mucosa mediated by IgE-driven Th2 immune responses. AR has a high global prevalence, ranging from 10% to 40% across regions [1]. It can occur at any age, although its incidence is particularly high among children and adolescents [2]. In childhood, the immune system is not fully mature, which may increase sensitivity to environmental allergens. During adolescence, immune activity is relatively high, daily activities expand, and the likelihood of allergen exposure increases, thereby promoting allergic responses. With the decline in environmental quality, the number of individuals affected by AR has continued to increase year by year [3]. AR not only causes physical discomfort but also imposes psychological and quality-of-life burdens [4]. Patients commonly experience nasal congestion, nasal itching, sneezing, rhinorrhea, and headache, which may impair olfaction and interfere with daily work and life [5]. Pharmacological and surgical treatments are commonly used for patients with mild to severe AR. At present, the main drugs used for AR treatment include glucocorticoids and antihistamines [6]. However, long-term use may cause local nasal mucosal injury, drowsiness, and other adverse effects. Surgical treatment can relieve nasal congestion by nerve ablation or turbinate reduction, but its limitations are also evident [7]. Because AR results from an excessive immune response to allergens, surgery cannot modify the underlying allergic predisposition, and postoperative recurrence is common [8]. Moreover, surgical risks, including nasal mucosal injury, nerve damage, and infection, should not be underestimated. Therefore, safer and more effective therapeutic strategies are still needed to reduce nasal mucosal injury while preserving nasal cavity integrity.

Extracellular vesicles (EVs) are lipid bilayer-enclosed vesicles released by cells. According to their diameters and biogenesis, EVs can be classified as exosomes, microvesicles, and apoptotic bodies [9]. Plant extracellular vesicles are nanoscale vesicles, typically 30–500 nm in diameter, secreted by plant cells [10]. These vesicles mediate intercellular communication between plants and animals, participate in pathogen defense, regulate plant innate immunity, and serve as carriers for mRNA, miRNA, bioactive lipids, proteins, and other therapeutic cargos in animal cells. They have been reported to exhibit anti-inflammatory, antitumor, anti-aging, and other biological activities [11]. Plant-derived extracellular vesicles retain biological functions associated with their source plants and can therefore act as natural therapeutic agents [12]. Compared with animal-derived exosomes, plant-derived vesicles show favorable safety, stability, and biocompatibility [13]. Increasing evidence has explored the role of exosomes in AR. For example, human mesenchymal stem cell-derived exosomes can regulate secretory protein expression in immune cells and epithelial tissues, reduce the number of Th2 cells, and inhibit Th2 differentiation, thereby alleviating AR symptoms [14]. However, most current studies have focused on animal-derived exosomes, and the effects of plant-derived extracellular vesicles on AR require further investigation.

*Angelica dahurica* is a tall perennial herb whose root is used medicinally and is recognized in China as a traditional medicinal and edible plant [15]. Previous studies have shown that active constituents of *Angelica dahurica*, including polysaccharides, volatile oils, and coumarins such as imperatorin and isoimperatorin, possess immunomodulatory and anti-inflammatory activities [16]. Imperatorin from *Angelica dahurica* can inhibit cancer growth through cell death receptor-mediated mechanisms [17]. In addition, the aqueous extract of *Angelica dahurica* has analgesic effects, and its polysaccharide fraction shows marked immune-enhancing activity [18]. *Angelica dahurica* is also an indispensable component of traditional Chinese medicine prescriptions used for AR treatment [19]. Nevertheless, few studies have investigated the effect of *Angelica dahurica*-derived exosome-like nanovesicles on AR. Therefore, in this study, we selected *Angelica dahurica*, a medicinal and edible Chinese herb with a favorable safety profile, as the raw material for isolating enriching exosome-like nanovesicles (ADELNs) and evaluating their therapeutic effect on AR.

Compared with nasal drops, nasal sprays, and oral administration, ultrasonic nebulization offers several advantages. Some patients cannot tolerate the transient irritation caused by nasal drops or sprays, while oral administration has a slower onset of action. Ultrasonic nebulization uses high-frequency ultrasound vibration to convert liquid into fine droplets [20]. During normal breathing, these droplets may facilitate local administration to the nasal mucosa and provide a potential approach for improving local exposure while avoiding the limitations associated with oral administration. Ultrasonic nebulization has attracted increasing attention for the treatment of airway inflammatory diseases, including pulmonary diseases and asthma [21]. Given the limited research on ultrasonic nebulization for AR, this study provides a new reference for treating AR through ultrasonic nebulized ADELNs.

## 2. Method

### 2.1. Isolation and Purification of Exosome-like Nanovesicles from Angelica dahurica

As described previously, exosome-like nanovesicles (ADELNs) were isolated from *Angelica dahurica* by differential centrifugation [22]. Briefly, Angelicae Dahuricae Radix (Song Cao Tang, Danbeier Biotechnology Co., Ltd., Tianjin, China) was pulverized using an ultrafine grinder (Guangzhou LeiMai Mechanical Equipment Co., Ltd., Guangzhou, China). *Angelica dahurica* powder was mixed with phosphate-buffered saline (PBS; Beijing Dingguo Changsheng Biotechnology Co., Ltd., Beijing, China) at a ratio of 1:8 (*w*/*v*, 1 g powder: 8 mL PBS) and thoroughly homogenized, followed by centrifugation at 4 °C (TGL-185, Hunan Pingfan Technology Co., Ltd., Changsha, China) at 2800× *g* for 30 min and then 11,000× *g* for 60 min to remove large impurities and fibers. The supernatant was further purified through a 0.45 μm aqueous membrane and subsequently centrifuged at 100,000× *g* for 60 min using an ultracentrifuge (Optima XE, Beckman Coulter, Brea, CA, USA). After the first ultracentrifugation, the supernatant was discarded, and the crude ADELN pellet was resuspended in an appropriate volume of PBS. The resuspended ADELNs were loaded onto a sucrose density gradient column composed of 15%, 30%, 45%, and 60% sucrose solutions and centrifuged at 100,000× *g* for 60 min. Sucrose density gradient ultracentrifugation was performed to further purify ADELNs from proteins, RNA, and other vesicles deposited in the previous step. All centrifugation steps were performed at 4 °C. The fraction between 30% and 45% sucrose, which has been previously reported to contain enriched plant-derived exosome-like nanovesicles, was collected and transferred to a centrifuge tube containing PBS [13]. ADELNs were collected by centrifugation at 100,000× *g* for 60 min and resuspended in PBS. Samples were stored short-term at 4 °C or rapidly frozen in liquid nitrogen and stored at −80 °C until analysis.

### 2.2. ADELNs Characterization

#### 2.2.1. Negatively Stained Transmission Electron Microscopy (TEM)

For negative staining and TEM analysis, 10 μL of sample was dropped onto a copper grid and allowed to settle for 1 min, after which excess liquid was removed with filter paper. Uranium acetate (10 μL) was then added dropwise to the copper grid for 1 min, and excess liquid was removed with filter paper. After drying at room temperature for several minutes, samples were imaged by electron microscopy at 80–120 kV (JEOL JEM-1400Plus, Tokyo, Japan) to obtain TEM images.

#### 2.2.2. Nanoparticle Tracking Analysis (NTA)

Nanoparticle tracking analysis (NTA) was performed using a NanoSight NS300 system (Malvern Panalytical, Malvern, Worcestershire, UK). Samples were diluted with sterile PBS to 10^7^–10^9^ particles/mL. The dilution ratio was determined according to pre-experimental optimization, and samples were filtered through a 0.22 μm membrane before detection. Standard operating procedures or manually defined test parameters were used. After samples were loaded into a quartz cuvette, Brownian motion trajectories were continuously recorded for 60 s in triplicate, and particle hydrodynamic diameter and concentration were calculated using NTA 3.4 software. D50, D90, and particle size distribution (bin width, 10 nm) were also calculated.

#### 2.2.3. Nanoparticle Size and Zeta Potential Analysis (DLS)

Nanoparticle size and zeta potential were analyzed using a Zetasizer Nano ZS90 system (Malvern Panalytical, UK). Samples were diluted with sterile PBS to 0.1–1 mg/mL according to the optimized pre-experimental concentration, filtered through a 0.22 μm membrane, and injected into a quartz cuvette for particle size analysis or a folded capillary electrophoresis cell for zeta potential analysis. Standard operating procedures or manually defined parameters were used. Z-average particle size and polydispersity index (PDI) were calculated based on intensity distribution statistics. Zeta potential was obtained by fitting electrophoretic mobility using the Smoluchowski model. Data were processed using Zetasizer Software 7.13.

#### 2.2.4. Lipid Extraction and TLC Analysis

Total lipids from ADELNs were extracted according to the Bligh and Dyer method [23]. ADELNs were dissolved in a chloroform/methanol mixture (2:1), and the mixture was thoroughly vortexed and mixed. The mixture was centrifuged at 2000× *g* for 10 min to achieve complete phase separation. The organic layer was collected and dried, and the precipitate was resuspended in chloroform. Lipid samples were separated on a silica gel thin-layer chromatography (TLC) plate using chloroform/methanol/acetic acid (190:9:1) as the developing solvent and visualized with 10% CuSO4 and 8% phosphoric acid reagent.

#### 2.2.5. Sodium Dodecyl Sulfate-Polyacrylamide Gel Electrophoresis (SDS-PAGE) and Agarose Gel Electrophoresis

Proteins extracted from ADELNs were separated by SDS-PAGE and stained with Coomassie brilliant blue for 30 min. Protein extraction was performed using a protein isolation kit (Beyotime Biotechnology, Shanghai, China). The protein concentration was 2 mg/mL, and 10 μL was loaded per well. Electrophoresis was performed at 80 V for 30 min and then at 120 V for 45 min until the bands were sufficiently separated. After staining, gels were destained with ethanol/glacial acetic acid/water (50 mL:100 mL:850 mL) for observation. RNA extracted from ADELNs was separated by agarose gel electrophoresis using an RNA extraction kit (Accurate Biotechnology Co., Ltd., Hefei, China). The RNA concentration was 500 ng/μL, and 2 μL of sample was loaded per well. RNA extracted from ADELNs was treated with or without RNase, and electrophoresis was performed on a 1.5% agarose gel at 150 V for 30 min. The gel was then observed using the Gel Doc XR+ Gel Documentation System (Bio-Rad, Hercules, CA, USA).

### 2.3. Cell Culture

Mouse mononuclear macrophage leukemia cells (RAW264.7) were donated by Dr. Shuya Huang (Second Hospital of Shandong University, Jinan, China). RAW264.7 cells were cultured in DMEM (Procell, Wuhan Procell Life Science & Technology Co., Ltd., Wuhan, China) supplemented with 10% FBS (ExCell Bio, Suzhou Ecosciences Biotechnology Co., Ltd., Suzhou, China) and 1% penicillin-streptomycin (Procell, Wuhan Procell Life Science & Technology Co., Ltd., Wuhan, China). All in vitro cell-based experiments, including the MTT assay, Neutral Red Experiment, ELISA, Atomization Stability Detection, Western blot and qRT-PCR, were carried out using *n* = 3 independent biological replicates.

### 2.4. MTT

Cell viability was assessed using the metabolic reduction assay based on 3-[4,5-dimethylthiazol-2-yl]-2,5-diphenyltetrazolium bromide (MTT) in living cells. Cells were seeded in 96-well plates at 1 × 10^5^ cells/well, cultured at 37 °C with 5% CO_2_ for 12 h, and then treated with ADELNs (0.001, 0.01, 0.1, 1, or 10 μg/mL) for 24 h. Absorbance at 570 nm was measured using a microplate reader (SpectraMax ABS, Oak Ridge, TN, USA) according to the manufacturer’s instructions (Solarbio, Beijing Solarbio Science & Technology Co., Ltd., Beijing, China).

### 2.5. Neutral Red Experiment

Cells were treated with blank medium, LPS (250 ng/mL; Sigma-Aldrich, St. Louis, MO, USA), or different concentrations of ADELNs combined with LPS for 24 h. After treatment, the original medium was discarded, 100 μL of 0.33% prewarmed neutral red dye was added, and cells were incubated at 37 °C for 2 h. After removal of the neutral red solution, cells were washed twice with PBS. Subsequently, 150 μL of neutral red extraction solution (ethanol:glacial acetic acid = 1:1) was added to each well. The 96-well plate was shaken for 10 min to fully resuspend the cells, and optical density was measured at 540 nm using a microplate reader (SpectraMax ABS, USA).

### 2.6. Enzyme Linked Immunosorbent Assay (ELISA)

RAW264.7 cells were seeded in 24-well plates (5 × 10^5^ cells/well). Cells were treated with blank medium, LPS (250 ng/mL), or different concentrations of ADELNs combined with LPS for 24 h, and cell supernatants were then collected. According to the manufacturer’s instructions, IL-6 and IL-1β levels (Dakewe Biotech Co., Ltd., Shenzhen, China) were measured using ELISA kits. NO content in cell supernatants was detected using an NO detection kit (Beyotime Biotechnology Co., Ltd., Shanghai, China).

### 2.7. Atomization Stability Detection

Medical/household nebulizers were purchased from Demi Medical Equipment Co., Ltd. (Guangzhou, China). The inhalation chamber consisted of four main parts: (1) an elbow pipe with an inner diameter of 20 mm; (2) a transparent PVC pipe, 5 cm in length and 20 mm in outer diameter; (3) a six-way tube with a 4 cm-long tube, 1 cm inner diameter, and upper vent of 20 mm inner diameter; and (4) six plastic straight-through pagoda joints (32 mm to 16 mm). To assess the stability of the nebulization device, ADELNs were nebulized for 5 min, samples were collected from each nebulization chamber, and miRNA and protein contents were measured before and after nebulization. The miRNA and protein contents of different ADELN concentrations before and after nebulization were measured using a SteadyPure small RNA extraction kit (Accurate Biotechnology Co., Ltd., Hefei, China) and a BCA protein quantification kit (Beyotime Biotechnology Co., Ltd., Shanghai, China), respectively. In addition, nebulization rate and efficiency were assessed. For a defined nebulization volume, the weight of the empty nebulizer cup before nebulization was recorded as W0, the weight of the nebulizer cup plus the liquid volume as W1, and the weight of the nebulizer cup after nebulization as W2. The time required for the complete nebulization of ADELNs was recorded as T. The nebulization rate was expressed as (W1 − W2)/(W1 − W0) × 100%, and nebulization efficiency was expressed as (W1 − W2)/T. Finally, nebulization uniformity was assessed by measuring weight changes in each nebulization chamber within a defined nebulization time.

### 2.8. Establishment the Mouse Model of AR Induced by Ovalbumin (OVA)

Six-week-old female specific-pathogen-free (SPF) C57BL/6 mice were purchased from Beijing Vital River Laboratory Animal Technology Co., Ltd. (Beijing, China). After arrival, mice were acclimatized for 7 days before the initiation of experiments. Mice were housed under standard conditions of humidity, room temperature, and light–dark cycles. Mice were provided with standard laboratory chow and sterile water ad libitum. All animal experiments complied with the ARRIVE guidelines and were performed in accordance with the U.K. Animals (Scientific Procedures) Act 1986 and associated guidelines, EU Directive 2010/63/EU for animal experiments, and the National Institutes of Health Guide for the Care and Use of Laboratory Animals (NIH Publication No. 8023, revised 1978). The animal experimental protocol complied with the Animal Management Rules of the Chinese Ministry of Health (Document No. 55, 2001) and was approved by the Institutional Animal Welfare and Ethics Committee of the Institute of Biology, Shandong Academy of Sciences (protocol code SWS20260311 and date of approval 11 March 2026). Mice were randomly divided into six groups (*n* = 6 per group) by random number generation: the control, AR model, sea salt spray control (SSS; TAI BANG, Zhuhai Nino Biotechnology Co., Ltd., Zhuhai, China), and the ADELN low-dose (L, 3.2 μg/mL), medium-dose (M, 16 μg/mL), and high-dose (H, 80 μg/mL) groups. The modeling method was modified based on previous studies [24]. On days 0, 7, and 14, mice in the AR, SSS, and ADELN groups were intraperitoneally injected with 200 μL of sensitization solution containing 250 μg OVA (Solarbio, Beijing Solarbio Science & Technology Co., Ltd., Beijing, China) and 5 mg aluminum hydroxide (Sinopharm Chemical Reagent Co., Ltd., Shanghai, China) dissolved in 1 mL normal saline for basic sensitization. Control mice received the same dose of normal saline. From days 21 to 27, nasal stimulation was performed using OVA (500 μg/20 μL) for the AR, SSS, and ADELN groups, with 10 μL administered into each nostril; control mice received the same volume of normal saline. Mice in the control, SSS, and ADELN groups were administered the corresponding treatment 30 min before nasal challenge. Specifically, 10 μL of SSS was sprayed into each nostril, the ADELN groups received nebulization for 5 min, and the control group received 5 min of normal saline nebulization. On day 28, behaviors were observed, and mice were sacrificed. The body weight of each individual mouse was monitored on days 0, 7, 14, 21, and 28 throughout the experimental period.

### 2.9. Nasal Symptom Assessment

Nasal rubbing and sneezing behaviors were assessed in a blinded manner. Nasal symptoms were recorded for 15 min after the final OVA nasal provocation and 1 h of rest. Sneezing and nose rubbing frequencies were independently recorded by two blinded observers.

### 2.10. Inflammatory Factor Analysis in Nasal Lavage Fluid (NALF) and Serum

Blood was collected from the tail vein, and mice were then sacrificed by cervical dislocation. Cold PBS (1 mL) was gently perfused from the trachea to the nasopharynx, and nasal lavage fluid was immediately collected. The collected nasal lavage fluid was centrifuged at 450× *g* for 7 min at 4 °C to remove impurities [25]. According to the manufacturer’s instructions, ELISA kits were used to detect the IL-6, IL-1β, and TNF-α levels (Dakewe Biotech Co., Ltd., Shenzhen, China) in the nasal lavage fluid supernatants. OVA-specific IgE (OVA-sIgE), histamine, IL-13, and IL-4 levels (Meibiao Biotechnology, Jiangsu Meibiao Biotechnology Co., Ltd., Yancheng, China) in the serum supernatants were detected according to the reagent manufacturer’s instructions.

### 2.11. Histological Analysis

The whole nasal cavities of the selected mice were fixed in 10% neutral buffered formaldehyde for one week, decalcified in 0.1 M EDTA buffer (Bio-solution, Suwon, Republic of Korea) for two weeks, and embedded in paraffin. Blocks were sectioned into 5 μm slices and stained with hematoxylin (Sigma-Aldrich, St. Louis, MO, USA) and eosin (Sigma-Aldrich, St. Louis, MO, USA) to analyze nasal mucosal thickness. The remaining slides were stained with toluidine blue dye (Wuhan Servicebio Technology Co., Ltd., Wuhan, China) to analyze mast cell infiltration. The slides were viewed using SaiViewer (version 3.0.1, Wuhan Servicebio Technology Co., Ltd., Wuhan, China) and analyzed using Image-Pro Plus (version 6.0, Media Cybernetics, Rockville, MD, USA).

### 2.12. The qRT-PCR Analysis of Nasal Mucosa Samples

The collected nasal mucosa was lysed in an appropriate amount of Trizol reagent (Sigma-Aldrich, St. Louis, MO, USA). The total RNA extraction kit was used to collect the total RNA, and complementary DNA (cDNA) was synthesized by reverse transcription using the Evo *M-MLV* reverse transcription kit (Accurate Biotechnology Co., Ltd., Hefei, China) according to the manufacturer’s instructions. The SYBR Green *Pro Taq* HS pre-mixed qPCR kit (Accurate Biotechnology Co., Ltd., Hefei, China) and real-time fluorescence quantitative PCR analyzer (Rotor-Gene Q, QIAGEN, Germany) were used for qRT-PCR. The sequence of PCR primers is listed in Table 1.

The relative expression of the three target genes was determined using the cycle threshold (2^−△△CT^) method and normalized to the β-actin level.

### 2.13. Western Blotting

Total protein was extracted from RAW264.7 cells, and cytoplasmic and nuclear proteins were separated using a nuclear-cytoplasmic separation kit (Beyotime Biotechnology Co., Ltd., Shanghai, China). Protein concentration was measured using a BCA kit. After gel electrophoresis, protein samples (20 μg) were transferred to PVDF membranes (Merck Millipore, Billerica, MA, USA). The following primary antibodies were added to the membranes: p65, phospho-p65, IκBα, phospho-IκBα, β-actin, and Lamin B1 (1:12,000 A19653; 1:15,000 AP1294; 1:4500 A24909; 1:1200 AP0707; 1:100,000 AC026; 1:85,000 AC057; ABclonal Technology Co., Ltd., Wuhan, China), followed by the appropriate secondary antibody (1:6000 AS014; ABclonal Technology Co., Ltd., Wuhan, China). Signals were detected using an enhanced chemiluminescence reagent (ECL; GE Healthcare, Chicago, IL, USA) and imaged using an Amersham Imager 600 system (GE Healthcare, Uppsala, Sweden). Protein bands were imaged using a gel imager (ChemiDoc XRS System, Bio-Rad, Hercules, CA, USA) and analyzed using ImageJ-win64 software.

### 2.14. Statistical Analysis

Results are expressed as mean ± standard deviation (SD). GraphPad Prism 9 software (GraphPad Software, San Diego, CA, USA) was used for graphical processing. The Shapiro–Wilk test was performed to assess data normality, and Levene’s test was used for homogeneity-of-variance evaluation. For multi-group comparisons, one-way ANOVA followed by Tukey’s post hoc test was applied to calculate the *p*-values for inter-group differences. *p* < 0.05 was considered statistically significant.

## 3. Results

### 3.1. Characterization of ADELNs

**Figure 1:**
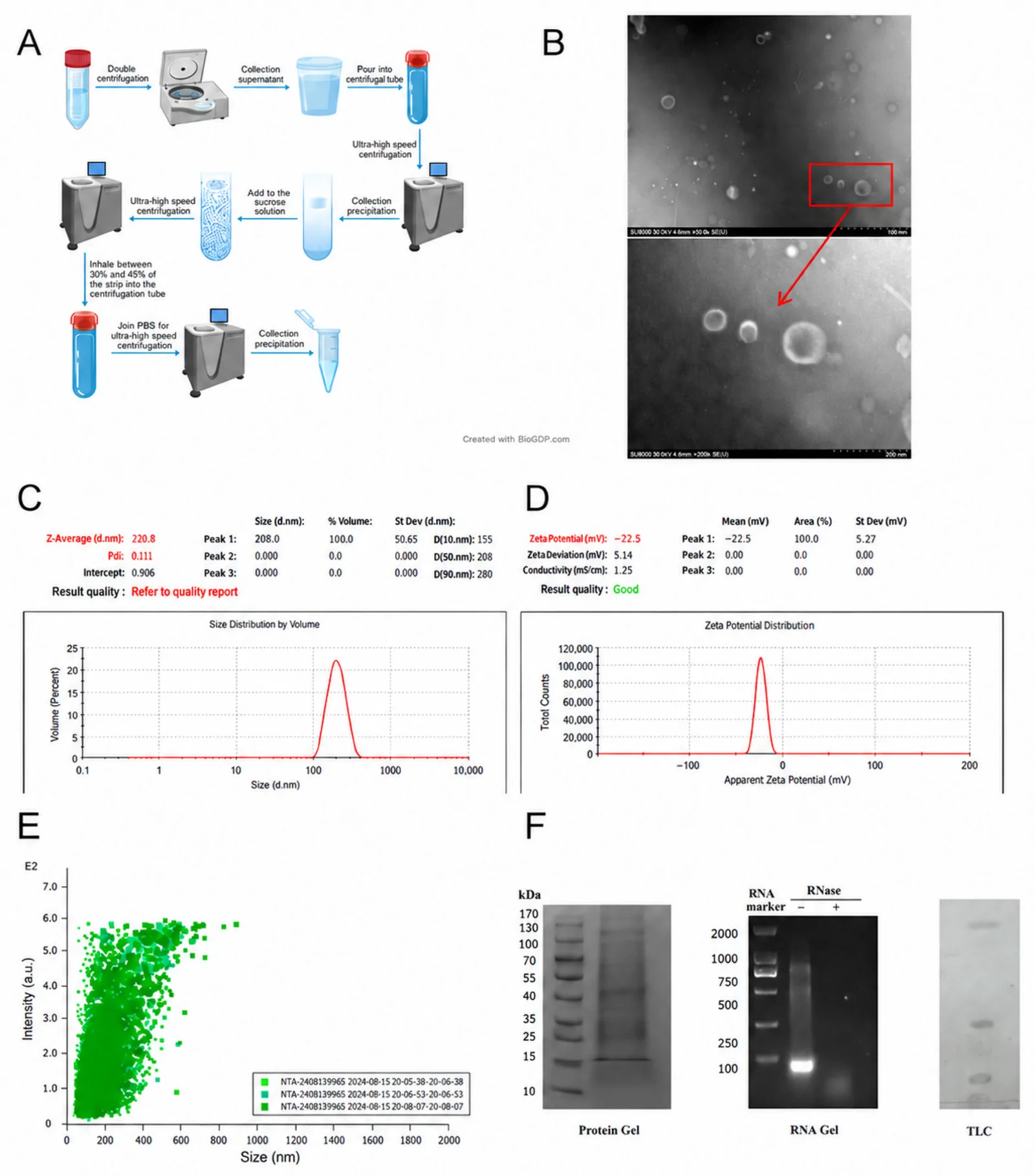
Characterization of ADELNs. (**A**) A schematic diagram of the extraction and purification process of exosomes from *Angelica dahurica* (Created with BioGDP.com). (**B**) Electron microscope image of ADELNs. (**C**) The average particle size of ADELNs obtained by DLS. (**D**) The potential of ADELNs obtained by DLS. (**E**) The particle size distribution of ADELNs obtained by NTA. (**F**) SDS-PAGE analysis of protein in ADELNs (left); agarose gel electrophoresis analysis of RNA in ADELNs (middle); TLC analysis of lipids in ADELNs (right).

The ADELN extraction process is shown in Figure 1A. NTA, TEM, DLS, and other techniques were used to analyze and characterize the ADELNs [26]. NTA and TEM showed that *Angelica dahurica*-derived vesicles exhibited a typical cup-shaped morphology (Figure 1B). The hydrodynamic diameter of ADELNs measured by DLS was 220.8 nm, with a polydispersity index (PDI) of 0.111, indicating a relatively narrow size distribution. The predominant size classes were 164.2, 190.1, 220.2, and 255.0 nm, with volume percentages of 18.4%, 22.3%, 20.7%, and 15.4%. Figure 1C indicates a relatively narrow size distribution. NTA analysis further confirmed the nanoscale size distribution of the ADELNs, which was consistent with the DLS results (Figure 1E). The zeta potential of the ADELNs was −22.5 mV (Figure 1D), suggesting the presence of stable negatively charged nanoscale vesicle-like structures. The particle-to-protein ratio was used as a means of comparing sample purity [27]. The particle concentration was 2.44 × 10^12^ ± 2.25 × 10^10^ particles/mL (Figure 1E), and the protein concentration was 2 mg/mL. The resulting particle-to-protein ratio was approximately 1.2 × 10^9^ particles/μg. SDS-PAGE analysis revealed that the ADELNs contained protein components mainly distributed in the range of approximately 15–25 kDa (Figure 1F, left). RNA analysis revealed that the ADELNs contained predominantly small RNAs (Figure 1F, middle). RNase treatment resulted in degradation of the extracted RNA, confirming the presence of RNase-sensitive nucleic acids associated with ADELNs. TLC revealed at least three abundant lipid components in ADELNs within the detection range (Figure 1F, right). These characterization results demonstrated that the isolated vesicles exhibited exosome-like features and supported the successful isolation of ADELNs.

### 3.2. Effect of ADELNs on the Viability of RAW264.7 Cells

**Figure 2:**
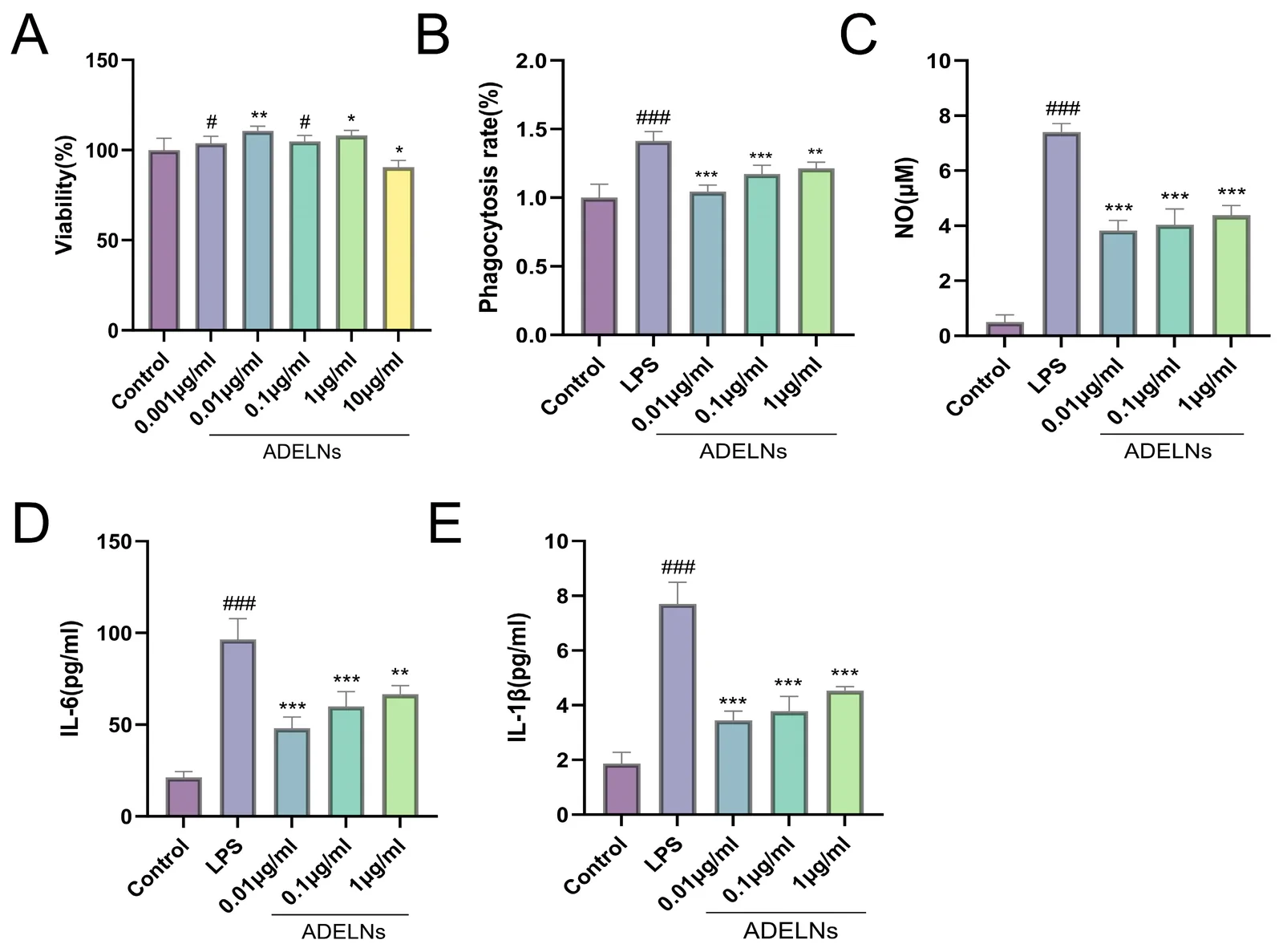
The effects of ADELNs on the content of NO, IL-6, IL-1β and TNF-α in LPS-induced inflammatory cells. (**A**) The effect of ADELNs on the viability of RAW264.7 macrophages. (**B**) The effect of ADELNs on LPS-induced endocytic activity in RAW264.7 cells. (**C**–**E**) The effect of ADELNs on the production of NO, IL-6 and IL-1β in LPS-induced RAW264.7 cells. Data are presented as mean ± SD; # *p* > 0.05 vs. Control, * *p* < 0.05, ** *p* < 0.01, *** *p* < 0.001, vs. LPS; ### *p* < 0.001, vs. Control.

To evaluate the cytotoxicity of ADELNs in RAW264.7 cells, cells were incubated with different concentrations of ADELNs for 24 h. The MTT assay results are shown in Figure 2A. Compared with the untreated control group, cell viability was significantly decreased when the ADELN concentration increased to 10 μg/mL, whereas no decrease in cell viability was observed after treatment with 0.001–1 μg/mL ADELNs. Because ADELN concentrations ranging from 0.001 to 1 μg/mL showed no obvious cytotoxicity, concentrations of 0.01–1 μg/mL were selected for subsequent experiments to ensure sufficient exposure and evaluate the concentration-dependent biological effects of ADELNs while avoiding the potential cytotoxic effects observed at higher concentrations (10 μg/mL).

### 3.3. ADELNs Reduce LPS-Induced Endocytic Activity in RAW264.7 Cells

To evaluate the effect of ADELNs on LPS-induced endocytic/lysosomal activity in RAW264.7 macrophages, cells were stimulated with 250 ng/mL LPS, as shown in Figure 2B. After cell adhesion, LPS (250 ng/mL) was added, and cells were incubated with ADELNs (0.01–1 μg/mL) for 24 h. Compared with the untreated control group, neutral red uptake in the LPS group increased by 41.35% (*p* < 0.001), indicating enhanced lysosomal/endocytic activity associated with macrophage activation. After ADELN treatment, endocytic activity decreased to varying degrees compared with the LPS group. These results indicate that ADELNs reduced the LPS-induced enhancement of neutral red uptake and macrophage activation-associated endocytic activity.

### 3.4. ADELNs Can Reduce the Content of NO, IL-6 and IL-1β in LPS-Induced Inflammatory Cells

To verify the anti-inflammatory activity of ADELNs, RAW264.7 cells were cultured in 24-well plates for 12 h and then treated with different concentrations of ADELNs combined with LPS for 12 h. NO, IL-6, and IL-1β levels in the cell supernatants were measured, and the results are shown in Figure 2C–E. After LPS (250 ng/mL) treatment, the NO, IL-6, and IL-1β levels were significantly higher than those in the control group (*p* < 0.001). After ADELN treatment, the NO, IL-6, and IL-1β levels were lower in all three ADELN concentration groups than in the LPS group. These results showed that ADELNs reduced LPS-induced inflammation in RAW264.7 cells.

### 3.5. Design of Mouse Atomization Chamber and Its Stability Analysis

**Figure 3:**
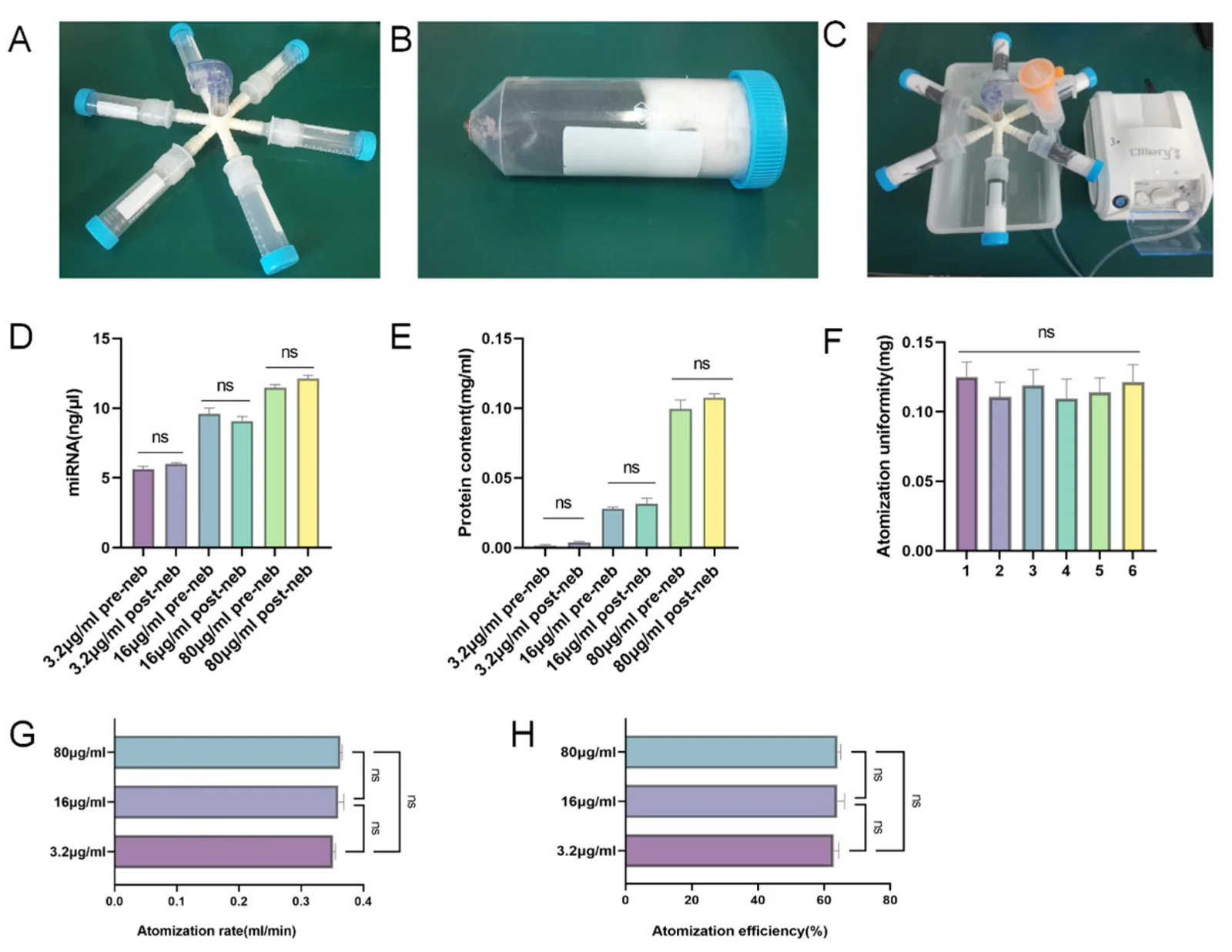
Stability analysis of ultrasonic atomization device. (**A**) Six-way pipe suction chamber for mice. The chamber consists of 4 parts (inlet elbow pipe, transparent PVC pipe, six-way pipe, straight pagoda joint). (**B**) Mouse fixed chamber. (**C**) Mouse nebulization display. (**D**) Comparison of miRNA content before and after atomization of different concentrations of ADELNs. (**E**) Comparison of protein content before and after atomization of different concentrations of ADELNs. (**F**) Homogeneity of atomization, that is, the weight comparison of each atomization chamber for five minutes of atomization. (**G**) Comparison of atomization rate of ADELNs with different concentrations. (**H**) Comparison of atomization efficiency of different concentrations of ADELNs. Data are presented as mean ± SD; ns, *p* > 0.05.

To establish a mouse model for evaluating ultrasonic nebulized administration, we developed an ultrasonic nebulization inhalation device specifically designed for nasal exposure in mice. The device consisted of an inhalation chamber and six mouse fixation chambers (Figure 3A). The inhalation chamber was composed of a six-way pipe, a transparent PVC pipe, an elbow pipe, six variable-diameter pagoda joints, and six branch pipes. The elbow pipe was connected to the nebulizer, and the six-way pipe was connected to the mouse fixation chambers through variable-diameter pagoda joints. A small hole was created in the wall of each variable-diameter joint to minimize pressure accumulation in the nebulization chamber. A 50 mL centrifuge tube was used as the mouse holding chamber. Mice were restrained with medical absorbent cotton to minimize movement and reduce its influence on inhaled dose. In addition, the tip of the centrifuge tube was removed to form a smooth-edged opening that could be easily positioned near the mouse nose (Figure 3B). Mouse nebulization is shown in Figure 3C. The elbow pipe above the inhalation chamber was connected to the nebulizer for nebulization. During nebulization, the six branches of the inhalation chamber were placed horizontally to ensure uniform dose delivery to each chamber. To confirm device stability, miRNA and protein contents were measured before and after nebulization (Figure 3D,E). The results indicated that the miRNA and protein contents of ADELNs remained stable across different concentrations (3.2, 16, and 80 μg/mL) before and after nebulization, suggesting that the device did not cause substantial loss or degradation of ADELNs. We further assessed nebulization uniformity, defined as the number of droplets reaching each nebulization chamber (Figure 3F). No significant difference was observed among chambers. Nebulization rate and efficiency were also analyzed (Figure 3G,H), and no significant differences were observed among the three ADELN concentrations, indicating that ADELN concentration had little effect on the nebulization efficiency or rate. Collectively, these results demonstrate that the mouse ultrasonic nebulization device had good stability.

### 3.6. ADELNs Can Alleviate the Nasal Symptoms of AR Mice and Reduce the Levels of Inflammatory Factors in Nasal Lavage Fluid and Serum

**Figure 4:**
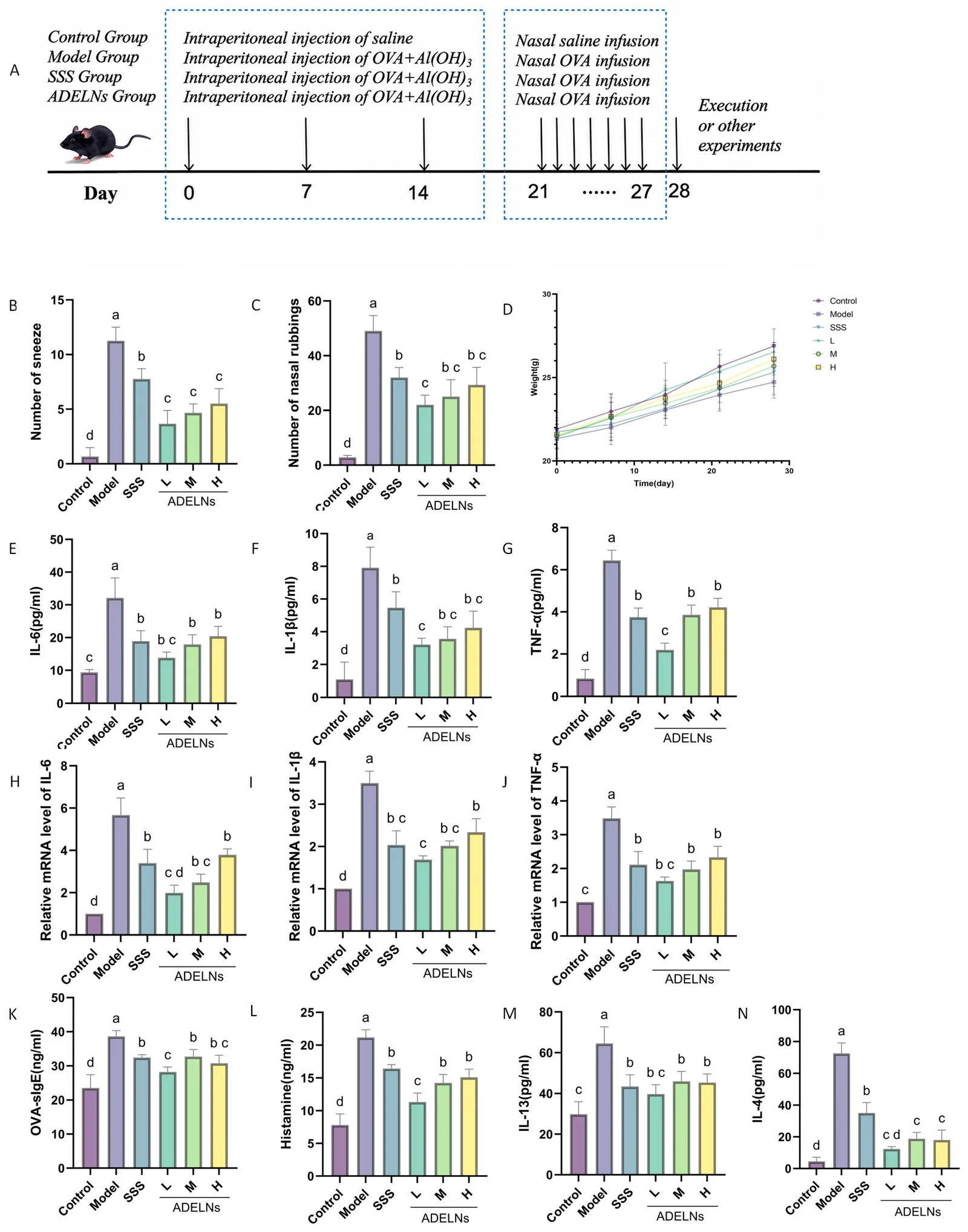
Effects of ADELNs on nasal symptoms and inflammatory factors in nasal lavage fluid and serum of AR mice. (**A**) The modeling process of AR mice. (**B**) The number of sneezes recorded after the last OVA stimulation and 1 h of rest in mice. (**C**) The number of nose scratches recorded after the last OVA stimulation and 1 h of rest in mice. (**D**) The body weight changes of mice on days 0, 7, 14, 21, and 28 during the whole modeling and administration period. (**E**–**G**) The contents of IL-6, IL-1β and TNF-α in nasal lavage fluid were measured. (**H**–**J**) The mRNA expression levels of IL-6, IL-1β and TNF-α in nasal mucosa of mice. (**K**–**N**) The contents of OVA-sIgE, histamine, IL-13 and IL-4 in serum = 6 mice per group; L, M, and H represent ADELN concentrations of 3.2, 16, and 80 μg/mL; Data are presented as mean ± SD, and statistical analyses were conducted using one-way ANOVA with Tukey’s post hoc analysis. Bars labeled with different letters indicate significant differences *p* < 0.05).

The modeling procedure for AR mice is shown in Figure 4A. Videos of sneezing and nose scratching were recorded for each group, and the number of sneezes and nose scratches were counted (Videos S1–S6, Behavioral observation video of different groups of mice.

As shown in Figure 4, sneezing (Figure 4B) and nose scratching (Figure 4C) were significantly increased in the model group. After SSS or ADELN treatment, these symptoms were alleviated to varying degrees, with the ADELN low-dose group showing the strongest effect; however, no significant difference in sneezing or nose scratching was observed between the SSS and model groups. Body weight changes were recorded throughout modeling and administration (Figure 4D). Nasal lavage fluid and serum were collected for further analysis. In nasal lavage fluid (Figure 4E–G), the IL-6, IL-1β, and TNF-α levels were significantly higher in the model group than in the control group (*p* < 0.001). After ADELN treatment, the IL-6, IL-1β, and TNF-α levels in the SSS, L, M, and H groups decreased to varying degrees, and the effect of ADELNs was more pronounced than that of SSS. Among the ADELN groups, the low-dose group showed the most significant effect (*p* < 0.001). These three inflammatory factors were also examined at the gene expression level in mouse nasal mucosa (Figure 4H–J), yielding results similar to those observed at the protein level. In serum (Figure 4K–N), the OVA-sIgE, histamine, IL-13, and IL-4 levels were significantly higher in the model group than in the control group (*p* < 0.01). After ADELNs or SSS treatment, the OVA-sIgE, histamine, IL-13, and IL-4 levels decreased to varying degrees, with low-dose ADELNs showing the most significant effect. Overall, these results indicate that ADELNs reduced the inflammatory factor levels in nasal lavage fluid and serum in the OVA-induced AR mouse model, thereby suppressing inflammation.

### 3.7. ADELNs Improves the Pathological State of AR Nasal Mucosa

**Figure 5:**
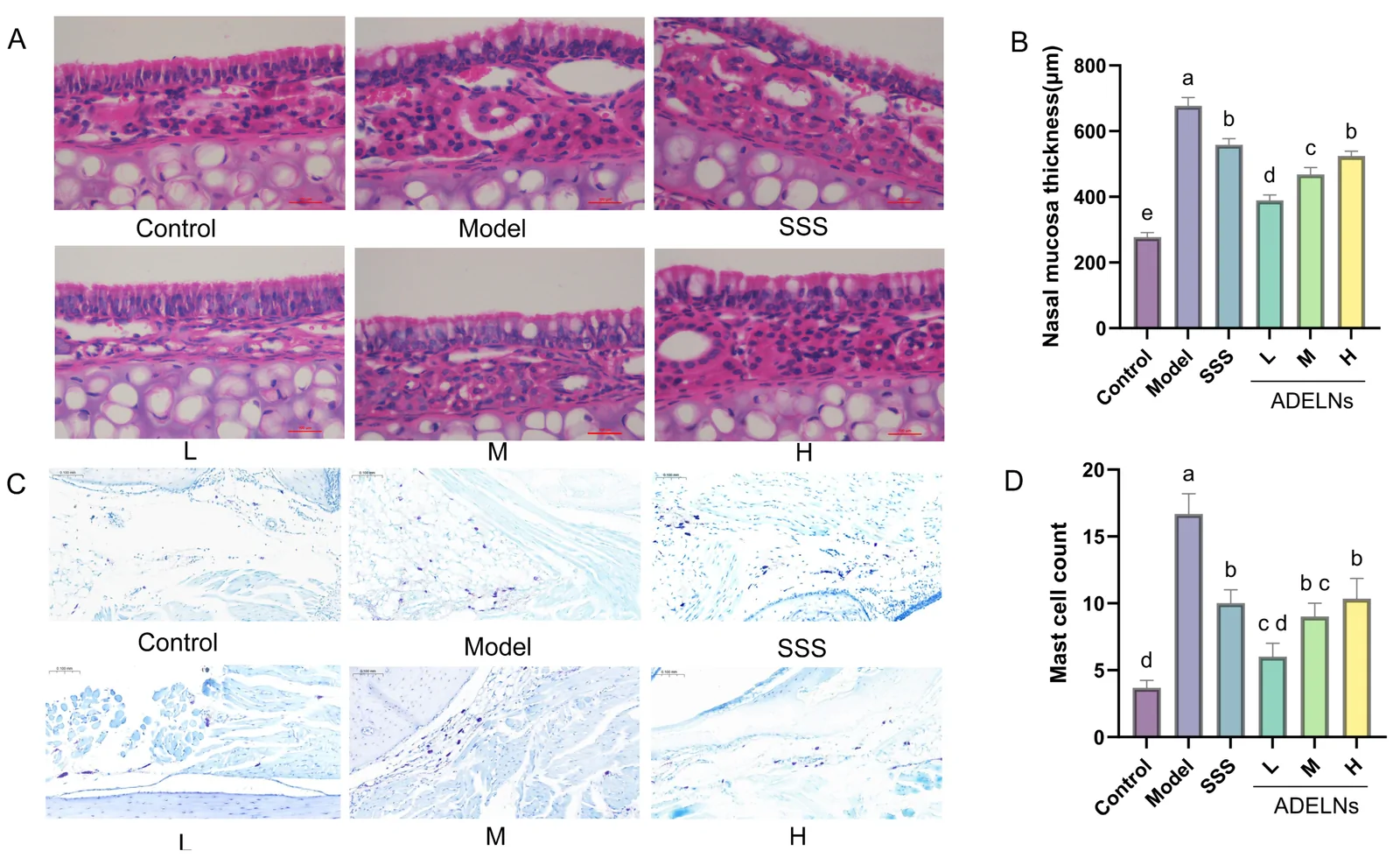
The effect of ADELNs on nasal mucosa pathology in AR mice. (**A**) H&E staining and thickness quantification of nasal mucosa. (**B**) Quantified nasal mucosa thickness. (**C**) Toluidine blue staining showing the mast cells (red arrows refer to mast cells). (**D**) The quantified number of mast cells. (*n* = 6; L, M, and H represent ADELN concentrations of 3.2, 16, and 80 μg/mL; data are presented as mean ± SD, and statistical analyses were conducted using one-way ANOVA with Tukey’s post hoc analysis. Bars labeled with different letters indicate significant differences *p* < 0.05).

Because AR symptoms are mainly localized in the nose, we examined pathological changes in nasal tissue sections from ADELN-treated AR mice using H&E staining (Figure 5). In the model group, nasal mucosal thickness increased due to inflammatory cell accumulation beneath the epithelium, ciliary arrangement became less compact and orderly, and vasodilation was more pronounced. After ADELN or SSS treatment, nasal mucosal thickness and ciliary organization improved compared with the model group (Figure 5A). Quantitative analysis showed that nasal mucosal thickness was significantly greater in the model group than in the control group and was significantly reduced after SSS or ADELN treatment. The low-dose ADELN group showed the most pronounced effect (Figure 5B). To further examine the type of infiltrating cells contributing to increased nasal mucosal thickness, mast cells were stained with toluidine blue (Figure 5C). The number of mast cells in the model group was significantly higher than that in the control group, as indicated by red arrows. After ADELN or SSS treatment, mast cell numbers decreased to varying degrees compared with the model group, and low-dose ADELNs showed the strongest effect in reducing mast cell infiltration in the nasal mucosa (Figure 5D). These results indicate that ADELNs reduced mast cell infiltration and improved the pathological state of nasal mucosa in AR mice.

### 3.8. ADELNs May Reduce NF-κB Pathway Activation in AR

**Figure 6:**
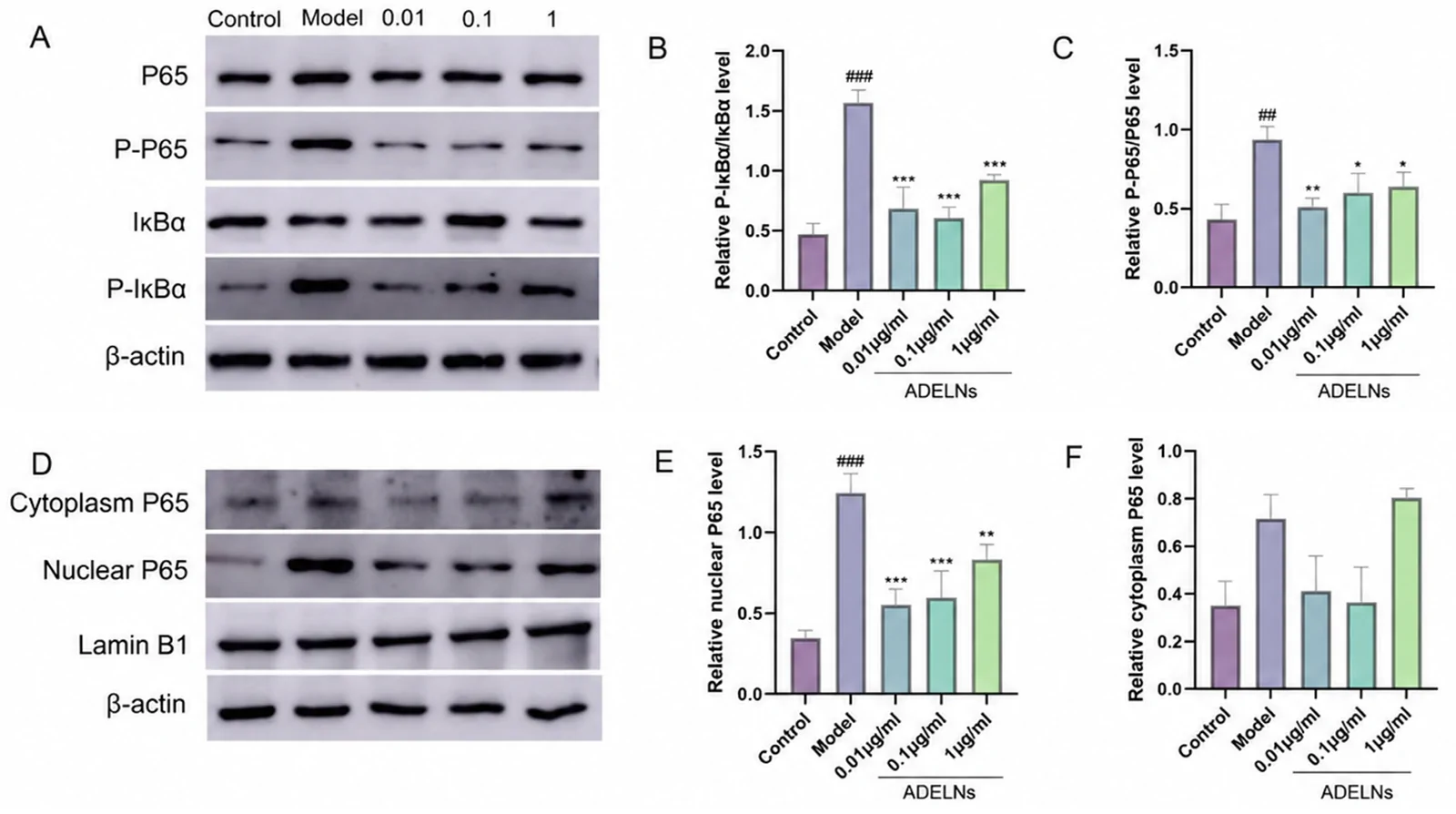
The effect of ADELNs on the NF-κB pathway. (**A**) The expression levels of P65, P-P65, IκBα, P-IκBα, β-actin. (**B**) The quantification of P-P65/P65 level. (**C**) The quantification of P-IκBα/IκBα level. (**D**) The expression levels of Cytoplasm P65, β-actin, Nuclear P65, Lamin B1. (**E**) The quantification of P65 level in the nucleus. (**F**) The quantification of P65 level in the cytoplasm. Data are expressed as mean ± SD; * *p* < 0.05, ** *p* < 0.01, *** *p* < 0.001 vs. Model; ## *p* < 0.01, ### *p* < 0.001, Unmarked, *p* > 0.05 vs. Control.

To evaluate the effect of ADELNs on the NF-κB pathway, we examined the NF-κB pathway-related proteins p65, phospho-p65, IκBα, and phospho-IκBα in cells (Figure 6A–C). Compared with the control group, the phospho-p65 and phospho-IκBα expression levels were significantly upregulated in the model group, and these increases were inhibited after ADELN treatment. To further verify NF-κB pathway activation, p65 levels in the nucleus and cytoplasm were also measured separately (Figure 6D–F). The results showed that p65 translocated from the cytoplasm into the nucleus, where it exerted its transcriptional regulatory function. Nuclear p65 levels were significantly increased in the model group, whereas ADELN treatment reduced the nuclear p65 levels compared with the model group, with the low-dose group showing the strongest effect after quantification. These results suggest that reduced NF-κB pathway activation may be involved in the anti-inflammatory effects of ADELNs.

## 4. Discussion

As a traditional medicinal and edible plant, *Angelica dahurica* is often used to dispel wind and unblock orifices in traditional treatment strategies for AR. Coumarins such as imperatorin and isoimperatorin in *Angelica dahurica* extracts have been reported to alleviate AR by inhibiting mast cell degranulation [28]. Based on this scientific rationale, we isolated extracellular vesicles from *Angelica dahurica* and analyzed their size distribution, concentration, and morphology by NTA, DLS, and TEM. The results showed that the average particle size of the cup-shaped vesicles was 220.8 nm, slightly larger than that of animal-derived exosomes, which may be related to their plant origin. The vesicles had a negative zeta potential, consistent with the surface charge characteristics of exosomes. Notably, the structural characteristics of extracellular vesicles isolated from *Angelica dahurica* were similar to those of exosomes from yam, papaya, and other plant sources, with average particle sizes generally ranging from 100 to 300 nm [29]. These vesicles also showed negative surface potentials and cup-shaped morphology, consistent with common features of plant-derived exosome-like nanovesicles. We further analyzed the major biomolecular components of ADELNs and found that they contained proteins, small RNAs, lipids, and other substances. These components may collectively contribute to the biological properties of ADELNs; however, the specific contribution of individual components remains to be elucidated. These components may act together to exert anti-inflammatory effects. Previous studies have suggested that proteins [30], small RNAs [31], and lipids associated with extracellular vesicles may participate in intercellular communication and immune regulation [32].

AR is an allergic disease characterized by chronic nasal mucosal inflammation and immune imbalance [33]. It is also one of the most common chronic diseases worldwide [34]. In the early stage of AR, antigen-presenting cells present allergens to T cells and induce T cell differentiation, triggering a series of immune responses [35]. Macrophages are important antigen-presenting cells and possess strong phagocytic capacity [36]. During AR-associated inflammation, macrophages eliminate allergens, apoptotic immune cells, and other substances while simultaneously secreting inflammatory factors such as IL-6, IL-1β, and TNF-α to participate in immune regulation [37]. NO is also an important signaling molecule secreted by macrophages. In the acute inflammatory phase, inducible nitric oxide synthase in macrophages synthesizes large or even excessive amounts of NO, thereby aggravating inflammation [38]. Therefore, inhibiting the activated macrophage production of NO and pro-inflammatory cytokines may help alleviate AR. Given the important role of macrophages in AR, we first selected macrophages to verify the anti-inflammatory activity of ADELNs. The results showed that macrophage lysosomal/endocytic activity and the levels of NO, IL-6, and IL-1β secreted by macrophages were significantly lower after ADELN intervention than in the model group, and the effect of ADELNs showed concentration dependence. Because macrophage phagocytic activity is generally positively associated with the degree of inflammation, these findings confirm that ADELNs can effectively alleviate macrophage lysosomal/endocytic activity and inflammatory factor release by downregulating LPS-induced inflammatory mediator production.

Nebulized inhalation is an effective approach for treating respiratory diseases [39]. Compared with oral administration, nasal drops, or topical application, ultrasonic nebulization may provide a potential approach for the local administration of ADELNs to the nasal cavity. Ultrasonic nebulization generates micron-sized aerosols through high-frequency vibration [40]. Compared with electronic nebulization, it produces finer and more uniform droplets, which may facilitate aerosol dispersion and nasal mucosal deposition [41]. Therefore, we selected ultrasonic nebulized inhalation as the administration route. To evaluate the feasibility of ultrasonic nebulized administration in mice, we developed a mouse nasal-only exposure nebulization device and first evaluated its stability [42]. Studies have shown that proteins and miRNAs are among the most important functional cargos in exosomes [43]. Their pivotal roles in inflammatory regulation and immune intervention have been demonstrated in numerous studies. Because detection technologies for proteins and miRNAs are relatively mature and widely used, we measured the protein and miRNA contents in ADELNs before and after nebulization to assess device stability. The results showed that the detectable protein and miRNA contents of ADELNs were maintained after nebulization, suggesting that the ultrasonic nebulization process did not cause substantial loss of these major molecular cargos. In addition, the self-designed nebulization chamber exhibited a relatively consistent distribution of nebulized liquid among different exposure compartments, supporting the feasibility of this system for animal experiments.

The main symptoms of AR include nasal itching, rhinorrhea, sneezing, and related manifestations [44]. These symptoms are also important criteria for evaluating successful establishment of an AR mouse model. In this study, behavioral observation showed that sneezing and nose scratching in AR mice were significantly improved after ADELN intervention compared with the model group. Previous studies have also shown that AR-related symptoms can seriously affect daily life [45]. To further clarify the intervention value of ADELNs, sea salt spray was used as a positive control. SSS is one of the most commonly used adjunctive treatments for AR. Compared with ordinary physiological saline, SSS has a slightly higher osmotic concentration and contains certain minerals, allowing it to remove allergens from the nasal mucosa, moisturize the nasal mucosa, and alleviate nasal symptoms [6]. Moreover, SSS lacks the side effects associated with other chemical drugs and does not interfere with immune function in mice, making it an appropriate positive control. The nasal mucosa is the primary site of AR, and the detection of inflammatory factors in nasal mucosa can directly reflect the effect of ADELNs. IL-6, IL-1β, and TNF-α are core pro-inflammatory cytokines in nasal mucosal inflammatory responses [46]. Our results showed that both protein and gene expression levels of IL-6, IL-1β, and TNF-α in the nasal mucosa of AR mice were significantly lower after ADELN intervention than in untreated AR mice. To more comprehensively assess the inflammatory status of mice, serum indicators were also analyzed. AR is essentially an IgE-mediated chronic noninfectious Th2-type inflammation of the nasal mucosa [47]. During inflammation, Th2 cells secrete core cytokines such as IL-4 and IL-13 to regulate immune balance. OVA is a common allergen used for AR mouse modeling. After exposure to OVA, the mouse immune system produces OVA-specific IgE, and OVA-IgE levels directly reflect the response of AR mice to allergens [48]. Histamine released during mast cell degranulation also directly corresponds to the inflammatory severity of AR symptoms [49]. After measuring key serum indicators, we found that ADELNs reduced OVA sensitization-induced increases in IL-4, IL-13, OVA-IgE, and histamine levels. Although the decreased levels of OVA-specific IgE and histamine suggest an attenuation of allergic inflammatory responses, whether ADELNs directly regulate IgE production or immune cell activation remains unclear. The reduction in these indicators may also result from the overall alleviation of OVA-induced inflammatory responses. Further studies investigating B-cell activation and Th2-related immune regulation are needed to clarify the underlying mechanisms. In addition, pathological changes in nasal mucosa provide a more intuitive representation of inflammation severity. H&E staining clearly showed nasal mucosal thickness in different groups. Mast cells and their released mediators, such as histamine, play important roles in allergic inflammation. In this study, toluidine blue staining was used to evaluate mast cell infiltration in nasal mucosa, while serum histamine levels were measured as an indicator associated with allergic responses. ADELN treatment reduced both mast cell infiltration and histamine levels in AR mice, suggesting the attenuation of mast-cell-associated allergic inflammatory responses. This effect may be associated with the biological properties of ADELNs and their diverse bioactive components, together with the potential of ultrasonic nebulization to facilitate local nasal delivery and improve the exposure of ADELNs to the nasal mucosa.

The NF-κB signaling pathway plays a central role in the inflammatory response of AR. Upon activation, IκB is phosphorylated and dissociates from NF-κB. Free NF-κB then enters the nucleus, binds to promoters of various pro-inflammatory genes, promotes inflammatory factor expression, and exacerbates nasal mucosal inflammation [50]. Previous studies have shown that natural compounds such as quercetin and apigenin can alleviate AR symptoms by regulating NF-κB signaling activity [51]. Therefore, the modulation of excessive NF-κB activation has been considered an important strategy for controlling inflammatory responses in AR. In this study, ADELN intervention significantly reduced the gene and protein expression levels of inflammatory factors, including IL-6, IL-1β, and TNF-α, in the RAW264.7 cell inflammation model. Furthermore, ADELN treatment was associated with reduced phosphorylation levels of IκB and p65 and decreased nuclear translocation of p65, suggesting that the modulation of NF-κB signaling may contribute to the anti-inflammatory effects of ADELNs. To further evaluate NF-κB pathway activation, p65 levels in the cytoplasm and nucleus were analyzed. The results showed increased nuclear accumulation of p65 in the model group, indicating enhanced NF-κB activation. After ADELN intervention, nuclear p65 accumulation was reduced, suggesting that decreased NF-κB pathway activation may be associated with the anti-inflammatory effects observed after ADELN treatment.

Interestingly, ADELNs did not exhibit a simple linear dose–response relationship, with the low-dose group showing relatively stronger effects in several evaluated parameters. This phenomenon may be associated with the complex biological characteristics of exosome-like nanovesicles. Unlike conventional drugs containing well-defined active molecules, ADELNs represent a multi-component biological system containing diverse cargos, including proteins, lipids, and small RNAs, which may exert distinct and synergistic biological activities. Changes in ADELN concentration may influence the relative contribution and interaction of different cargos, thereby resulting in a non-linear biological response. Therefore, the stronger effects observed at lower concentrations may reflect the complex regulatory properties of multi-component nanovesicle systems rather than a conventional concentration-dependent pharmacological effect. Further investigation of cargo composition and functional interactions will help elucidate the mechanisms underlying the dose–response characteristics of ADELNs.

Overall, this study demonstrates that *Angelica dahurica*-derived extracellular vesicle-like nanovesicles delivered through ultrasonic nebulization can alleviate OVA-induced allergic inflammatory responses in mice. These findings highlight the potential of plant-derived nanovesicles as a natural therapeutic platform for inflammatory diseases and provide a foundation for further mechanistic and translational studies. In summary, this study explored the potential application of *Angelica dahurica*, an edible and medicinal plant, in the treatment of AR. For the first time, *Angelica dahurica*-derived exosome-like nanovesicles were applied to AR through ultrasonic nebulization, innovatively combining ultrasonic nebulization technology with plant exosome-like nanovesicle delivery. This approach may facilitate the local nasal administration of ADELNs and provides a potential strategy for exploring plant-derived nanovesicles in AR treatment. It also provides new ideas for research on exosome-like nanovesicles derived from edible and medicinal plants. Although this study confirmed the alleviating effect of ADELNs on AR and explored part of its mechanism, several limitations remain. First, although ADELN treatment was associated with reduced NF-κB activation, the current study did not establish a direct causal relationship between NF-κB regulation and the therapeutic effects of ADELNs. Future studies using pathway-specific activation, inhibition, or rescue strategies will be necessary to further clarify the molecular mechanisms involved. Second, ADELNs contain diverse biological cargos, including proteins, lipids, and small RNAs; however, the specific components responsible for their biological activities remain unidentified. Comprehensive cargo profiling combined with functional validation will be important for identifying key active molecules and understanding their contributions. Third, although ultrasonic nebulization was demonstrated as a feasible administration approach for ADELNs in this mouse model, aerosol characteristics, actual deposited dose, nasal distribution, and biodistribution were not determined. Future translational studies should further evaluate particle-based dose characterization, aerosol delivery efficiency, and tissue distribution to establish a more precise relationship between administered dose and therapeutic response. In addition, comparisons with crude *Angelica dahurica* extract and vesicle-depleted fractions will be valuable for distinguishing vesicle-associated effects from soluble plant-derived components. Furthermore, although the mouse nebulization system provides a useful experimental platform for evaluating nasal delivery, its applicability to human intranasal administration requires further investigation.

## 5. Conclusions

In this study, ADELNs were successfully isolated and comprehensively characterized. ADELNs were administered to mice through a self-designed ultrasonic nebulization system. ADELNs attenuated LPS-induced inflammatory responses in vitro and alleviated allergic symptoms and pathological alterations in AR mice. ADELN treatment was associated with reduced IκB and p65 phosphorylation and decreased NF-κB nuclear translocation, suggesting that the modulation of NF-κB signaling may contribute to the anti-inflammatory effects of ADELNs. These findings provide preliminary evidence supporting ADELNs as a potential approach for alleviating allergic rhinitis-associated inflammation.

## Figures and Tables

**Table 1:** Primer sequences for quantitative real-time PCR.

Gene	Primer-F (5′–3′)	Primer-R (5′–3′)
β-actin	ACCTTCTACAATGAGCTGCGT	TACATGGCTGGGGTGTTGAAG
IL-6	TGATGGATGCTACCAAACTGGA	TCTCTCTGAAGGACTCTGGCT
IL-1β	TCCAGGATGAGGACATGAGCAC	AACGTCACACACCAGCAGGAAT
TNF-α	ACTCCAGGCGGTGCCTATGT	GTGAGGGTCTGGGCCATAGAA

## Data Availability

The data used and/or analyzed during the current study are available from the corresponding author on reasonable request.

## Supplementary Materials

The following supporting information can be downloaded at: https://www.mdpi.com/article/10.3390/pharmaceutics18091193/s1, Video S1: Sneezing and nose-scratching behavior in mice in the control group; Video S2: Sneezing and nose-scratching behavior in mice in the model group; Video S3: Sneezing and nose-scratching behavior in mice in the SSS group; Video S4: Sneezing and nose-scratching behavior in mice in the L group; Video S5: Sneezing and nose-scratching behavior in mice in the M group; Video S6: Sneezing and nose-scratching behavior in mice in the H group.

## References

[1] Wise S.K., Damask C., Roland L.T., Ebert C., Levy J.M., Lin S., Luong A., Rodriguez K., Sedaghat A.R., Toskala E. (2023). International Consensus Statement on Allergy and Rhinology: Allergic Rhinitis—2023. Int. Forum Allergy Rhinol..

[2] Agüero C.A., Sarraquigne M.P., Parisi C.A., Mariño A.I., López K., Menéndez Porfirio B., Sasia L., Lozano A., Bovina Martijena M.D.P., Gervasoni M.E. (2023). Allergic rhinitis in pediatrics: Recommendations for diagnosis and treatment. Arch. Argent. Pediatr..

[3] Czech E.J., Overholser A., Schultz P. (2024). Allergic rhinitis. Med. Clin. N. Am..

[4] Pagel J.M.L., Mattos J.L. (2024). Allergic rhinitis and its effect on sleep. Otolaryngol. Clin. N. Am..

[5] Iinuma T., Kiuchi M., Hirahara K., Kurita J., Kokubo K., Yagyu H., Yoneda R., Arai T., Sonobe Y., Fukuyo M. (2022). Single-cell immunoprofiling after immunotherapy for allergic rhinitis reveals functional suppression of pathogenic TH2 cells and clonal conversion. J. Allergy Clin. Immunol..

[6] Bernstein J.A., Bernstein J.S., Makol R., Ward S. (2024). Allergic rhinitis: A review. JAMA.

[7] Tosca M.A., Trincianti C., Naso M., Nosratian V., Ciprandi G. (2024). Treatment of allergic rhinitis in clinical practice. Curr. Pediatr. Rev..

[8] Klimek L., Mullol J., Ellis A.K., Izquierdo-Domínguez A., Hagemann J., Casper I., Davis A., Becker S. (2024). Current management of allergic rhinitis. J. Allergy Clin. Immunol. Pract..

[9] Pegtel D.M., Gould S.J. (2019). Exosomes. Annu. Rev. Biochem..

[10] Kimiz-Gebologlu I., Oncel S.S. (2022). Exosomes: Large-scale production, isolation, drug loading efficiency, and biodistribution and uptake. J. Control. Release.

[11] Batrakova E.V., Kim M.S. (2015). Using exosomes, naturally-equipped nanocarriers, for drug delivery. J. Control. Release.

[12] Salunkhe S., Dheeraj, Basak M., Chitkara D., Mittal A. (2020). Surface functionalization of exosomes for target-specific delivery and in vivo imaging & tracking: Strategies and significance. J. Control. Release.

[13] Cao M., Diao N., Cai X., Chen X., Xiao Y., Guo C., Chen D., Zhang X. (2023). Plant exosome nanovesicles (PENs): Green delivery platforms. Mater. Horiz..

[14] Fang S.B., Zhang H.Y., Wang C., He B.X., Liu X.Q., Meng X.C., Peng Y.Q., Xu Z.B., Fan X.L., Wu Z.J. (2020). Small extracellular vesicles derived from human mesenchymal stromal cells prevent group 2 innate lymphoid cell-dominant allergic airway inflammation through delivery of miR-146a-5p. J. Extracell. Vesicles.

[15] Hu J., Xu F., Zhu L., Cui Y., Au R., Li Y., Tong Y., Shen H. (2025). *Angelica dahurica* polysaccharides ameliorate colitis by reducing the restriction of gut microbiota-derived imidazole propionate on PPAR-γ signaling activation. Phytother. Res..

[16] Wei W., Wu X.W., Deng G.G., Yang X.W. (2016). Anti-inflammatory coumarins with short- and long-chain hydrophobic groups from roots of *Angelica dahurica* cv. Hangbaizhi. Phytochemistry.

[17] Zheng Y.M., Shen J.Z., Wang Y., Lu A.X., Ho W.S. (2016). Anti-oxidant and anti-cancer activities of *Angelica dahurica* extract via induction of apoptosis in colon cancer cells. Phytomedicine.

[18] Zhu C., Wang M., Guo J., Su S.L., Yu G., Yang Y., Zhou Y., Tang Z. (2022). *Angelica dahurica* extracts attenuate CFA-induced inflammatory pain via TRPV1 in mice. Evid. Based Complement. Altern. Med..

[19] Kung Y.Y., Chen Y.C., Hwang S.J., Chen F.P. (2006). The prescriptions frequencies and patterns of Chinese herbal medicine for allergic rhinitis in Taiwan. Allergy.

[20] Naidu H., Kahraman O., Feng H. (2022). Novel applications of ultrasonic atomization in the manufacturing of fine chemicals, pharmaceuticals, and medical devices. Ultrason. Sonochem..

[21] Xu X., Wang Y., Luo X., Gao X., Gu W., Ma Y., Xu L., Yu M., Liu X., Liu J. (2023). A non-invasive strategy for suppressing asthmatic airway inflammation and remodeling: Inhalation of nebulized hypoxic hUCMSC-derived extracellular vesicles. Front. Immunol..

[22] Seo K., Yoo J.H., Kim J., Min S.J., Heo D.N., Kwon I.K., Moon H.-J. (2023). Ginseng-derived exosome-like nanovesicles extracted by sucrose gradient ultracentrifugation to inhibit osteoclast differentiation. Nanoscale.

[23] Zhou X., Xu S., Zhang Z., Tang M., Meng Z., Peng Z., Liao Y., Yang X., Nüssler A.K., Liu L. (2024). Gouqi-derived nanovesicles (GqDNVs) inhibited dexamethasone-induced muscle atrophy associating with AMPK/SIRT1/PGC1α signaling pathway. J. Nanobiotechnol..

[24] Choi S., Jung M.-A., Hwang Y.-H., Pyun B.-J., Lee J.Y., Jung D.H., Ji K.-Y., Kim T. (2021). Anti-allergic effects of Asarum heterotropoides on an ovalbumin-induced allergic rhinitis murine model. Biomed. Pharmacother..

[25] Shin S.H., Ye M.K., Lee D.W., Che M.H. (2020). Immunomodulative effects of Chamaecyparis obtusa essential oil in mouse model of allergic rhinitis. Molecules.

[26] Jiang S., Li H., Zhang L., Mu W., Zhang Y., Chen T., Wu J., Tang H., Zheng S., Liu Y. (2025). Generic Diagramming Platform (GDP): A comprehensive database of high-quality biomedical graphics. Nucleic Acids Res..

[27] Webber J., Clayton A. (2013). How pure are your vesicles?. J. Extracell. Vesicles.

[28] Shi H., Chang Y., Feng X., Yang G., Zheng Y., Zheng Q., Zhang L., Zhang D., Guo L. (2022). Chemical comparison and discrimination of two plant sources of Angelicae dahuricae Radix, *Angelica dahurica* and *Angelica dahurica* var. formosana, by HPLC-Q/TOF-MS and quantitative analysis of multiple components by a single marker. Phytochem. Anal..

[29] Hwang J.H., Park Y.S., Kim H.S., Kim D.H., Lee S.H., Lee C.H., Lee S.H., Kim J.E., Lee S., Kim H.M. (2023). Yam-derived exosome-like nanovesicles stimulate osteoblast formation and prevent osteoporosis in mice. J. Control. Release.

[30] Choi D.S., Kim D.K., Kim Y.K., Gho Y.S. (2013). Proteomics, transcriptomics and lipidomics of exosomes and ectosomes. Proteomics.

[31] Liu S.S., Zha Z., Li C., Li C.Y., Wang L. (2025). The mechanism of exosomes of BMSCs modified with Bu Shen Yi Sui capsule in promoting remyelination via regulating miR-15b/Wnt signaling pathway-mediated differentiation of oligodendrocytes. J. Ethnopharmacol..

[32] van Niel G., D’Angelo G., Raposo G. (2018). Shedding light on the cell biology of extracellular vesicles. Nat. Rev. Mol. Cell Biol..

[33] Hao Y., Yang Y., Zhao H., Chen Y., Zuo T., Zhang Y., Yu H., Cui L., Song X. (2025). Multi-omics in allergic rhinitis: Mechanism dissection and precision medicine. Clin. Rev. Allergy Immunol..

[34] Broide D.H. (2010). Allergic rhinitis: Pathophysiology. Allergy Asthma Proc..

[35] Gómez F., Rondón C., Salas M., Campo P. (2015). Local allergic rhinitis: Mechanisms, diagnosis and relevance for occupational rhinitis. Curr. Opin. Allergy Clin. Immunol..

[36] Maurer M., Köberle M., Metz M., Biedermann T. (2019). Mast cells: Promoters of health and modulators of disease. J. Allergy Clin. Immunol..

[37] Cruse G., Bradding P. (2016). Mast cells in airway diseases and interstitial lung disease. Eur. J. Pharmacol..

[38] Miyasaka N., Hirata Y. (1997). Nitric oxide and inflammatory arthritides. Life Sci..

[39] Maselli D.J., Keyt H., Restrepo M.I. (2017). Inhaled antibiotic therapy in chronic respiratory diseases. Int. J. Mol. Sci..

[40] Tao W., Lai Y., Zhou X., Yang G., Wu P., Yuan L. (2025). A narrative review: Ultrasound-assisted drug delivery: Improving treatments via multiple mechanisms. Ultrasonics.

[41] Hao F., Bai Y., Xie X., Yuan T., Wei Y., Xiong Q., Gan Y., Zhang L., Zhang Z., Shao G. (2022). Phenotypic characteristics and protective efficacy of an attenuated Mycoplasma hyopneumoniae vaccine by aerosol administration. Vaccine.

[42] Tang Y., Qian J., Ding M., Ding R., Yang P., Dou Y. (2025). Nebulized MSC exosomes promote the transdifferentiation of transitional state cells against acute lung injury through STAT3/Krt8/AQP5 axis. Eur. J. Pharm. Biopharm..

[43] Nazri H.M., Greaves E., Quenby S., Dragovic R., Tapmeier T.T., Becker C.M. (2023). The role of small extracellular vesicle-miRNAs in endometriosis. Hum. Reprod..

[44] Bai X., Liu P., Shen H., Zhang Q., Zhang T., Jin X. (2022). Water-extracted Lonicera japonica polysaccharide attenuates allergic rhinitis by regulating NLRP3-IL-17 signaling axis. Carbohydr. Polym..

[45] Zhang J.J., He X.C., Zhou M., Liu Q.D., Xu W.Z., Yan Y.J., Ruan Y. (2023). Xiao-qing-long-tang ameliorates OVA-induced allergic rhinitis by inhibiting ILC2s through the IL-33/ST2 and JAK/STAT pathways. Phytomedicine.

[46] Lichenstein R., Giudice E.L. (2000). Nasal wash technique for nasal foreign body removal. Pediatr. Emerg. Care.

[47] Jafari M., Hjalmarsson E., Hellkvist L., Paziou E., Karlsson A., Kumlien Georén S., Cardell L.O. (2025). Persistent off-season dysregulation of memory B cell subsets in allergic rhinitis. Clin. Transl. Allergy.

[48] Lv R., Shan J., Sun A., Xing Z., Xu Q., Shao Q., Li H. (2025). Research progress of anti-IgE treatment for allergic rhinitis. Am. J. Otolaryngol..

[49] Zhao M., Zhang H., Liu Z., Liu J., Xie B., Zeng L., Wang X., Shu Q., Tang P., Mo L. (2025). Dynactin subunit 1 facilitates mast cell degranulation to drive food allergy pathogenesis. Immunol. Lett..

[50] Kang C., Li X., Liu P., Liu Y., Niu Y., Zeng X., Zhao H., Liu J., Qiu S. (2023). Tolerogenic dendritic cells and TLR4/IRAK4/NF-κB signaling pathway in allergic rhinitis. Front. Immunol..

[51] Li H., Zhang H., Zhao H. (2023). Apigenin attenuates inflammatory response in allergic rhinitis mice by inhibiting the TLR4/MyD88/NF-κB signaling pathway. Environ. Toxicol..

