# Supplementary material for: Angelica dahurica Exosome-like Nanovesicles Delivered by Ultrasonic Nebulization Alleviate Allergic Rhinitis and Modulate NF-κB Signaling
Source: Pharmaceutics. 2026 Sep 21;18(9):1193. doi: 10.3390/pharmaceutics18091193 (PMC13610228; doi:10.3390/pharmaceutics18091193)
Supplement: Supplementary file 1 [file pharmaceutics-18-01193-s001.zip › Video description.pdf]

Videos (S1–S6): Records of sneezing and nose scratching in different groups of mice

S1. The sneezing and nose scratching of mice in control group.

S2. The sneezing and nose scratching of mice in model group.

S3. The sneezing and nose scratching of mice in group SSS.

S4. The sneezing and nose scratching of mice in group L.

S5. The sneezing and nose scratching of mice in group M.

S6. The sneezing and nose scratching of mice in group H.
